# The Duties of the Hour

**Published:** 1882-08

**Authors:** C. Pearson

**Affiliations:** President


### THE DUTIES OF THE HOUR.

AN ADDRESS DELIVERED BEFORE THE INTERNATIONAL HAHNEMANNIAN ASSOCIATION AT INDIANAPOLIS, JUNE 13th, 1882.

By C. Pearson, M. D., President.

"In a science in which the welfare of mankind is concerned, any neglect to make ourselves masters of it becomes a crime."—*Hahnemann.*

These were noble words and nobly uttered, and while Carroll Dunham was willing to accord to every physician the right of private judgment or "liberty of medical opinion and action," he at the same time wished him not to lose sight of the fact that he assumed a "great responsibility."

When a prominent personage in the prime of life succumbs to disease, or its injudicious management, how often do we hear the expression, that all that medical skill and science could do, had been tried and failed; nothing is more common than this, and yet nothing as a general thing is more untrue.

In a majority of cases the only thing that would have proved efficacious was not resorted to at all. "I will save the life of my patient," says the advocate of freedom of medical opinion, "and I care not how it is done." This, at first sight, seems to be commendable, but is it true? Very rarely, for when those who are most ready to make this statement are advised to resort and adhere to Homœopathy as taught by its founder, they obstinately refuse and their patients die from neglect to comply with the only principles nature has provided for their recovery. And yet we hear them exclaim: "Would *you* let your patients die for the sake of adhering to a principle?" "Yes," I reply, "if die they must, rather than kill them for the lack of principle."

When a reform is greatly needed, it is thought a reformer usually appears, and seldom was a reform in anything more implicitly demanded than in medicine when, at the beginning of the present century, Hahnemann commenced to promulgate the true doctrine of therapeutics. But this was so far in advance of the age and so contrary to popular sentiment that, like all other reforms that have blest the world, it was derided and rejected. In order to overcome this prejudice, it was thought best by some of his so-called followers to compromise to some extent with his opposers, and this spirit of compromise then set up has never ceased, but, on the contrary, has become bold, defiant and aggressive; which is usually the case when any attempt is made to compromise truth with error.

At a meeting held in New York on the 89th anniversary of the birth of Hahnemann, 10th of April, 1844, to organize the American Institute of Homœopathy, very little of this disposition to compromise was manifested; the object seemed to be a union for the sake of strength and for the best interests of our school; hence, it was resolved that

"WHEREAS, a majority of allopathic physicians continue to deride and oppose the contributions to the materia medica that have been made by the homœopathic school; that one of the objects of the Institute shall be, to restrain physicians from pretending to be competent to practice Homœopathy, who have not studied it in a careful and skillful manner."

And at the second session it was resolved not to admit any one as a member who could not sustain an examination before the Board of Censors, on the theory and practice of Homœopathy. From this it would appear that the great object in organizing the American

Institute was to promote the cause of Homœopathy. But how does that Institute stand to-day in reference to this matter? Not one word is said about any knowledge of Homœopathy being a necessary qualification for membership.

Hahnemann, at an advanced period of his life, asserted that he could count on the fingers of one hand all his faithful disciples, and though we think a greater number may be found to-day, still, it cannot be denied that his true followers have not increased in the same ratio as those whom he denounced as the "new mongrel sect." One who ever since the organization of this association has been the most noisy and vituperative in regard to it, in an article published in the November, 1881, number of the *Medical Times*, says: "That the early homœopath, the 'International' is swiftly going to the wall, is demonstrated when we compare the relative increase of the two parties; at the first quarter of this century 100 per cent. were 'high;' at this date only one and one-half per cent. are of the original stock." In this same journal, which has ceased to fly the homœopathic flag at its mast-head, the editor says: "We are something more than homœopathic. While we practice homœopathically, we profess to have sense enough not to attempt to apply the principle where it does not belong." In an article published in the October 26th number of the *Medical Counsellor*, for 1881, the writer, who styles himself one of the oldest members of the homœopathic school, after advocating a union of the two schools of medicine, in speaking of the doctrine of dynamization, says: "The like of which, from a homœopathic point of view (!), for bold absurdity, pure fictitiousness and absolute unreasonableness, has seldom been witnessed in medical history, certainly not in our day." To this distinguishing feature, if not cardinal principle of Hahnemann's Homœopathy, this same writer applies such mild and complimentary adjectives as "visionary," "pernicious," "ridiculous and untenable," "extravagant and unjustifiable," "dangerous doctrine," "dynamization craze," "singular medical delusion," etc., and boasts that the large class to which he belongs are unbelievers; says they are not only unbelievers, but they *know* that dynamization has no lot or part with Homœopathy; that they *know* it was only a fanciful creation of Hahnemannn, "that every day we allow this empirical method to be taught at our colleges, we are *acting a lie;*" "every day that we listen to reports of these nondescript dynamic cases at the meetings of our societies and publish them as homœopathic without protest, we are acting *a lie*." A man once insisted that he had seen the devil, whom he described as having large ears, a long tail and in every respect resembling a great ass, but it was afterwards ascertained that he had only been frightened at his own shadow.

It is said that dealers in stock and in fish, become so impregnated with the odor of their business as to be impervious to the perfume of a flower, and those who speak so glibly of lies, are usually so filled with *their subject* as to be oblivious to everything else.

Now, it becomes important to know what proportion of the so-called homœopathic practitioners of the country these writers represent. Their effusions are published in the journals with scarcely a protest or comment, unless it come from a member of this association.

It is barely possible that these windy disciples of Hippocrates have no influence and represent no one but themselves; that all their accumulation of gas only requires a few doses of *Carb veg.*, or *Lycopodium*, and yet if they do not represent the masses, why are they, without a word of reproof, permitted from month to month and from year to year to vilify and falsify the plain teaching of Hahnemann. Political papers usually represent the principles of their party; in Theology, the church literature is usually the criterion by which we judge of their belief: very few so-called religious papers would publish Paine's "Age of Reason," and it is *painful* to know that greater heresies in regard to medicine are permitted to find a place in our literature. Only think of the *Christian Advocate* publishing, without a protest, Col. Ingersoll's lecture on the "Mistakes of Moses." Don't you suppose there would be some exceptions taken to it by some of the subscribers who supported that paper: and when all other remedies had failed would there not be likely to be another organization formed, probably an "International," adhering to orthodox principles? For in that case many would be likely to read the mistakes of Moses, who knew very little of Moses himself, and in this case many read the denunciations of Hahnemann's teachings, who never read his *Organon* in their lives, or if so they probably lacked the capacity to comprehend or to indorse it.

In an editorial in the *Hospital Gazette*, an allopathic medical journal published in New York, under date of April 25th, 1878, the editor after saying that Homœopathy has died by committing suicide, refers to resolutions passed by the homœopathic society of that city as follows:

"The homœopathic society of this city has declared that it will no longer 'obstruct science,' or make itself the jest and amusement of a laughing world, and it has formally announced its intention in the future to use any medicine which experience has proven to be useful, whether it operate in accordance with the rule *similia* or of *contraria*, etc. In short, to use what medicine they please without reference to rule or doctrine; and this is now, and always has been, the precept and practice of the school of medicine from which, for the sake of gain, in order that they might profit by a stupid but popular delusion, they had formally separated themselves. In admitting this they have virtually, so far as they and their followers are concerned, terminated the existence of Homœopathy. As to the number of deserters, those gentlemen who have taken the lead in firing upon, and hauling down their own flag, say that not three genuine homœopathists can be found in this city, probably not one (rather rough on some of the members of this association), not so many as were required to save Sodom and Gomorrah." Then he proceeds to denounce them, does not wish to own them or to be considered as belonging to the same family with them; hopes in looking for a new name they will not call themselves doctors, thinks eclectics or noodles would do better.

Smythe, in his work on "Medical Heresies," after quoting from numerous writers of the homœopathic school, to show they have abandoned every cardinal principle of Hahnemann's Homœopathy (and there is not and never was any other), in referring to the preamble and resolutions adopted as the platform of the International Hahnemannian Association, says, "The formation of this association and the adoption of this platform of principles is a return to the pure, inflexible, dogmatic Homœopathy of Hahnemann."

This is true, and, from a conservative standpoint, well expresses the sole object of those who instituted this movement. They beheld this retrograde tendency on every hand; from our journals and from our colleges they witnessed it in the striking out of the word Homœopathy in relation to the qualification for membership in the American Institute, and from some of the journals of the country. They became painfully conscious of it from the crude prescribing and consequent failures of so-called homœopathic physicians growing out of this "liberty of medical opinion and action." They looked across the water to the home of Hahnemann and Bœnninghausen, and saw that just in proportion as the principles of these veterans were abandoned, just in proportion had Homœopathy become unpopular. They saw the *Organon* of the former, around which the true men of the past were wont to stand as a wall of adamant, denounced and spit upon and strange gods instituted in its place. In view of these facts it was thought necessary to organize the International Hahnemannian Association, to demonstrate that the Homœopathy of Hahnemann had still a local habitation and a name, and to defend and maintain the principles which, as a homœopathist, he taught.

That this was the great object, end and aim, the preamble and resolutions adopted at our first meeting fully testify, while the necessity for the existence of such an association is demonstrated by the calumny heaped upon us by many of the physicians and journals of the drifting wing of the homœopathic school. These resolutions have been denounced as un-Hahnemannian and non-homœopathic, together with other charges equally false and, if possible, equally foolish.

This declaration of principles claims first that the "*Organon* of the healing-art, as promulgated by Samuel Hahnemann, is the only reliable guide in therapeutics," and it is marvelous how any one at all familiar with the teachings of the "master" can deny this; and yet it has been denied and its meaning been perverted and misrepresented. We have in this been accused of ignoring our works on materia medica and practice as guides; but that no such construction was ever intended to be put upon this language, perhaps no honest man believes. As the *Organon* preceded our other works, of course it was, or should have been, their "only reliable guide," and that it clearly teaches that Homœopathy consists in the law of the similars in part, no one will deny; but that it consists wholly in this no one who accepts our first proposition for a moment believes, for if so, then Hahnemann originated nothing but the name, the law having been known to others centuries before his time; and it had also been demonstrated that crude drugs prescribed according to it were not only inexpedient, but injurious, and for this reason both the law and the practice fell into disrepute.

But we declare further that the system of Hahnemann requires more than the law—that the single remedy is necessary. In paragraph 169 of the *Organon* we read, "It may easily occur, on examining a disease for the first time, and also in selecting for the first time the remedy that is to combat it, that the totality of the symptoms of the disease is found not to be sufficiently covered by the morbific symptoms of a single medicine, and that two remedies dispute the preference as to the eligibility in the present instance, the one being homœopathic to one part of the disease, and the other still more to another. It is then by no means advisable, after using the preferable of the two remedies, to take the other without examination, because the medicine given as the inferior of the two, under the change of circumstances, may not be proper for the remaining symptoms, in which case it follows that a suitable homœopathic remedy should be selected for the new set of symptoms in its stead."

No mixing of remedies is advised here, no alternating or rotating, but each medicine is to be given separately and singly, because of its adaptability to existing symptoms.

And not only is the single remedy advised but the "minimum dose." In paragraphs 279 and 280 we are told, "The dose of the homœopathic remedy can never be sufficiently small so as to be inferior to the power of the natural disease which it can, at least partially extinguish and cure, provided it be capable of producing only a small increase of symptoms immediately after it is administered." "This incontrovertible axiom, founded upon experience, will serve *as a rule by which the doses of all homœopathic medicines, without exception are to be attenuated to such a degree that after being introduced into the body they shall merely produce an almost insensible aggravation of the disease.* It is of little import whether the attenuation goes so far as to appear almost impossible to ordinary physicians whose minds feed on no other ideas but what are gross and material, all their arguments and vain assertions will be of little avail when opposed to the dictates of unerring experience." And not only are the single remedy and minimum dose advised, but this must be of the "dynamized drug." This appears to be plainly set forth in paragraph 16 of the *Organon*, where it is said, "The vital principle as a spiritual dynamis cannot otherwise be assailed and affected than in a (dynamic) spiritual manner; neither can such morbid disturbances, or, in other words, such diseases be removed by the physician except in like manner by means of the spiritual (dynamic virtual) countervailing agency of the suitable medicines acting upon the same vital principle." * Whatever exceptions may

---

* This controversy between the vitalists, or spiritualists, and the materialistic school, is as old as Athenaus (A. D. 50); it was again revived by Stahl in the 17th century. Each party has its advocates to-day as it had eighteen hundred be taken to this language of Hahnemann as to a vital force and spiritual property in drugs, it is very evident that the disease-producing, as well as the disease-curing agent is invisible or spirit-like, and this is probably all that he claimed, that this principle is not imparted to the drug by trituration and succussion, but only developed and set free by this process, being latent in the drug itself, seems to be most plausible. That neither the law of the similars, the single remedy or the minimum dose of the dynamized drug alone constitute the system of Homœopathy, is so plainly taught by Hahnemann that it would seem to be a useless waste of time to discuss it in the presence of those who are his followers in fact as well as in name. He says on page 630 of the Lesser Writings: "The medicine does not accomplish its object by means of quantity but by potentiality and quality (*dynamic fitness homœopathy*)."

That numbers of professed homœopathists not only violate these tenets but largely repudiate them, and that an effort has been made on the part of such physicians to unite the homœopathic with the allopathic school, we have already shown, and in view of these facts we resolve that the time has fully come when legitimate Hahnemannian homœopathists should publicly disavow all these innovations referred to. We declare that the mixing or alternating of two or more medicines is not Homœopathy as Hahnemann taught it, and he probably knew what he wished to say as well as those who think they know what he should have said. On these two points it is certainly not necessary to dwell, as every candid reader of his works must know that he plainly taught the use of one single, simple substance at a time, one remedy for one class of symptoms, another for another, after the former had expended its action. In regard to his directions in reference to the dose and its repetition, Dr. Dunham once said, "Not pretending that we do not often, through errors in judgment infringe them, we are sure that whenever we do so, misfortune follows, and that in proportion to our faithfulness, so is our success." * * * *

"The prejudice in favor of large and many doses is a relict of past ages, when the practitioner was paid not for his skill and professional

---

years ago. Hahnemann might be said to have belonged to the former. It is useless to contend about what we can *know* little or nothing, but one thing we do know, that whereas medicines will not act on the lifeless body, the life principle, whether it be called vital, dynamic, spiritual, or what you please, must be the medium through which they do act on the general system.

services, but for the *medicines* he furnished—a barbarous usage, which, along with slavery, we received from our British progenitors."

Why should we forget this and remember only what the same writer said in reference to "freedom of medical thought and action?" Why should we remember that Hahnemann cured a patient with the tincture of Bryonia, and forget that after he had discovered a surer and better way he condemned this treatment as unsafe and unreliable?

"In non-surgical cases we disapprove of medicated topical applications and mechanical appliances as being also non-homœopathic."

This, like all the rest of our resolutions, is Hahnemann's teaching. Let objectors make their fight with him, as some of them are honorable enough to do, and not with us.

In paragraph 203 of the *Organon* he says, "Every external treatment of a local symptom whose aim is to extinguish it on the surface of the body without curing the internal miasmatic disease, such, for example, as that of destroying a psoric eruption on the skin by means of ointments, healing up a chancre by the use of caustic, destroying the granulations of sycosis by ligature, excision or the application of a hot iron is not only useless, but injurious." * * * * "This is the most criminal practice physicians can adopt." "We cannot recognize as homœopathic such treatment as suppresses symptoms by the primary or toxical action of the drug."

Whoever, therefore, suppresses for the time being pain with morphine, intermittents with quinine, constipation with podophyllin, etc., is prescribing by contraries to all intents and purposes, and every supposed cure thus effected is brought about in precisely the same manner that allopaths are now, and have been for centuries, effecting theirs; if it be homœopathic for one class of physicians it is so for all, and he is the best homœopath who exhibits the largest powders and pills. Now we have no sympathy with such treatment, and do not wish to hold ourselves or Homœopathy responsible for its many failures.

We have already referred to the denunciation of Hahnemann and his teachings by self-styled homœopathists, and that we regard all such as recreant to the best interests of Homœopathy is, to say the least, "drawing it mild."

For the purpose of promoting these sentiments, in the month of June, at the city of Milwaukee, in 1880, this International Hahnemannian Association was organized, which now, at the expiration of the second year of its existence, numbers over sixty members. We do not claim that this list comprises all the Hahnemannians in the world; we hope there are others who will still join us, and I would suggest that Article III of the By-laws be so amended that a two-thirds majority may elect, instead of its having to be made unanimous, as is now the case. Some members may object to applicants for personal reasons, and these objections should not be allowed to have a controlling influence in this association. The question to be considered is: How does the applicant stand homœopathically? Does he indorse our platform of principles, and practice what he professes? Is he uncompromising in his adherence to Homœopathy as Hahnemann taught it? If so, as we are not exclusively a moral reform, but a *medical* reform society, we most respectfully ask him to join and help us, taking it for granted that none but gentlemen will apply, and that they are therefore morally eligible.

Another section I would suggest in our By-laws, or the adoption of a resolution to the same effect, that inasmuch as our association is international in its character, that foreign members, for all officers of the association, be permitted to vote by proxy.

It was not the original intention in organizing this association, to be antagonistic to any other. If any refuse to join us because in sentiment we may differ from them, it proves nothing except that they differ from us and from the inductive doctrine of Hahnemann. That there is between the homœopathic and the allopathic schools, an impassible barrier, no one can deny, the latter removing effects by palliative contraries, the former removing causes by similars. That the latter have within the last quarter of a century in a great measure discarded the large saddle-bags and doses of former years, is doubtless greatly to be attributed to the reform that Homœopathy has inaugurated; at the same time, while the quantity of their drugs has been greatly diminished, their potency has been correspondingly increased.

The tinctute of *Nux vomica* has given place to the deadly *strychnia*, gum opium to morphine, and Peruvian bark to *quinia;* and while we now hear little said of calomel, this poisonous metal, the hydra-headed hydrargyrum, is given in no less than thirty compounds.

And while with their capsules, their granules and sugar-coated pills, they make a crude outward imitation of Homœopathy, the enemy is only concealed in a Trojan horse, and when smuggled into the stomach will do its destructive work all the same. Did Homœopathy consist only in small doses as weighed in allopathic scales, then indeed are these men practicing homœopathically, but as we have shown that neither small doses of crude drugs nor the law of the similiars, or both these together constitute Homœopathy, no one who prescribes in this way can be accredited with practicing it whether he claims to belong to the regular, irregular or defective school; and those who thus prescribe are no more homœopathic with, than without the name: for here is the border-land where we separate, here the Rubicon crossed by Hahnemann after half a century's experimenting with crude drugs, here he bade them a final farewell, and here his true followers part company with the drug school, take up his line of march, not being restricted to the necessarily circumscribed investigations of one lifetime, however long and arduous, but disposed to still pursue his researches in the direction he pointed out.

If, instead of advancing with us, others should tarry by the way or turn backwards to grope amongst the exploded systems, falsely so-called of the past, it is their privilege, but let them first renounce the name of Hahnemann and discard all connection with his school.

We have no disposition to conceal the fact that of late years this divergence in our ranks has been going on to such an extent that to-day there is less similarity between the Hahnemannian and the eclectic branches of the homœopathic school in the management of the sick, than there is between the latter and the more advanced allopaths; in fact, their cures, where they effect any, are usually brought about in the same manner by the primary action of the drug. This dangerous and delusive practice of treating diseases as though they were local, of lopping off branches while the roots remain, of suppressing effects without regard to causes, of silencing the voice of pain with narcotics and anæsthetics, cause the patient and his friends to congratulate themselves on his improvement, and so he continues to improve till he dies. This deceptive custom—for it does not deserve the name of science or system—of treating organs as though they had no relation to the general system, and that each must necessarily have its special physician, has misled the community and filled the world with humbugs and charlatans. Quite recently a case came under my observation where in an attack of pleurisy the physician resorted to hypodermic injections of morphine, and when soon afterwards he was consulted as to the patient's chances for recovery, replied that it was very difficult to tell now, as he was under the influence of an opiate; before the doctor's arrival the case was doubtless less complicated. Is it any wonder then that those who believe that Hahnemann taught another and better way, should feel no disposition to be held responsible for the numerous failures that have always attended, and must forever attend, such treatment? For where this so strikingly assimulates, can we expect the results to be widely different? for so long as oil and water refuse to unite; so long as heat and cold continue to be opposite extremes; so long as light and darkness or truth and falsehood remain antagonistic, so long both in theory and in practice will the homœopathic differ from the allopathic school.

I would further suggest that Article I, of our By-laws, be so changed that the meetings of this Association may not be dependent in any way on those of the American Institute, and as we do not propose to be held responsible for much of the practice indorsed by a majority of its members, I can see no good reason why we should longer continue our connection with it. Of course, I do not speak officially in this matter, but only as an individual, and as such, have no hesitation in declaring that if the heresies advocated by some of its leading members are henceforth to become its governing principles, I, for one, am willing to follow or to lead in the direction I have suggested. "If this be treason, make the most of it." But it is not treason; it is loyalty to principle, loyalty to science and loyalty to pure, legitimate Homœopathy.

In an article published in the March, 1882, number of the *North American Review*, on the "Fallacies of Homœopathy," by Prof. Palmer, amidst a great mass of falsehood and misrepresentation, we are pleased to find this spasm of truth: "One believing in the efficacy of infinitesimals and in the injurious effects of medicines in crude forms and sensible doses, could not consent, with any regard to the supposed interests of his patient, to the administration of the larger doses. If, for the purpose of securing patronage, the homœopathist pretends to a superior system in which he does not believe, and to a better practice which he does not follow, he is a charlatan and a pretender, unworthy of confidence or honorable associations."

Do our *liberal* homœopathic friends like the colors in which these allopathic painters, with whom they would affiliate, have drawn them? Are they willing to

"Walk under their huge legs and peep about
To find themselves dishonorable graves?"

Then they must not complain of the treatment they receive. An honest man will ever respect honesty in others, and any attempt on the part of members of either school to unite them on any middle ground will, and of necessity ought forever to, be a failure. The difference is too great, the chasm too broad, to ever be bridged; contraries *or* similars, not contraries and similars, for both cannot be true; neither can truth be found between them, and while it may be that

"Truth crushed to earth will rise again,"

it may not do so in one age or generation, and it is better to prevent it from being crushed than to attempt its resurrection afterwards.

For this, the International Hahnemannian Association has been organized, and to this end let us, like a band of brothers, devote our best energies, laying aside all personal differences and minor considerations,

"All trivial fond records,
All saws of books, all forms, all pressures past
That youth and observation copied there;
That this commandment all alone may live
Within the book and volume of the brain,
Unmixed with baser matter."

